# Chromosome-Level Genome Assembly of the Blacktail Brush Lizard, *Urosaurus nigricaudus*, Reveals Dosage Compensation in an Endemic Lizard

**DOI:** 10.1093/gbe/evad210

**Published:** 2023-12-06

**Authors:** Elizabeth Davalos-Dehullu, Sarah M Baty, Robert N Fisher, Peter A Scott, Greer A Dolby, Adrian Munguia-Vega, Diego Cortez

**Affiliations:** Centro de Ciencias Genómicas, Programa de Biología de Sistemas, UNAM, Cuernavaca, Morelos, Mexico; Baja GeoGenomics Consortium; Baja GeoGenomics Consortium; School of Life Sciences, Arizona State University, Tempe, Arizona, USA; Western Ecological Research Center, U.S. Geological Survey, San Diego, California, USA; Natural Sciences Collegium, Eckerd College, St Petersburg, Florida, USA; Baja GeoGenomics Consortium; Department of Biology, University of Alabama at Birmingham, Alabama USA; Baja GeoGenomics Consortium; Conservation Genetics Laboratory, The University of Arizona, Tucson, Arizona, USA; Applied Genomics Lab, La Paz, Baja California Sur, Mexico; Centro de Ciencias Genómicas, Programa de Biología de Sistemas, UNAM, Cuernavaca, Morelos, Mexico; Baja GeoGenomics Consortium

**Keywords:** chromosome-level genome assembly, squamates, *Urosaurus nigricaudus*, Baja California Peninsula, endemism

## Abstract

*Urosaurus nigricaudus* is a phrynosomatid lizard endemic to the Baja California Peninsula in Mexico. This work presents a chromosome-level genome assembly and annotation from a male individual. We used PacBio long reads and HiRise scaffolding to generate a high-quality genomic assembly of 1.87 Gb distributed in 327 scaffolds, with an N50 of 279 Mb and an L50 of 3. Approximately 98.4% of the genome is contained in 14 scaffolds, with 6 large scaffolds (334–127 Mb) representing macrochromosomes and 8 small scaffolds (63–22 Mb) representing microchromosomes. Using standard gene modeling and transcriptomic data, we predicted 17,902 protein-coding genes on the genome. The repeat content is characterized by a large proportion of long interspersed nuclear elements that are relatively old. Synteny analysis revealed some microchromosomes with high repeat content are more prone to rearrangements but that both macro- and microchromosomes are well conserved across reptiles. We identified scaffold 14 as the X chromosome. This microchromosome presents perfect dosage compensation where the single X of males has the same expression levels as two X chromosomes in females. Finally, we estimated the effective population size for *U. nigricaudus* was extremely low, which may reflect a reduction in polymorphism related to it becoming a peninsular endemic.

**Significance**

The *Urosaurus nigricaudus* genome assembly is highly contiguous, well-annotated, and very syntenic with other squamates. This genome will be an invaluable resource for those elucidating evolutionary processes that have shaped peninsular biodiversity as well as in understanding genomic evolution across Lepidosauria/Tetrapoda.

## Introduction

Advancements in technology and more affordable costs for DNA sequencing and assembly have led to an increase in reference genomes for nonmodel organisms. Over the last 6 years, the number of reference genomes for reptiles has significantly grown. From 2018 to May of 2023, 113 reference genomes for Crocodylia (1 genome), Testudines (31 genomes), and Lepidosauria (81 genomes) were added to the National Center for Biotechnology (NCBI) database, bringing the total number of reptile reference genomes available to 127. However, this number still pales in comparison with the number of reference genomes available for other amniote groups, such as mammals (585 genomes) and birds (840 genomes), despite reptiles having a higher number of species (∼11,820 reptiles, ∼10,906 birds, and ∼6,615 mammals, according to The Reptile Database, Birds of the World, and the Mammal Diversity Database, respectively). From these 127 reptile reference genomes, only 37 are chromosome-level assemblies.

Reptiles are crucial models for understanding evolution and speciation owing to their diversity of unique genomic features, morphological traits, lifestyles, sex determination systems, venom evolution, coloring patterns, thermal regulation, and metabolic features (Chang, et al. 2009; Fry, et al. 2009; Janes, et al. 2010; Alfoldi, et al. 2011; Casewell, et al. 2012; Olsson, et al. 2013; Shine 2013; Tollis, et al. 2014; King and Lee 2015; Deakin and Ezaz 2019; Mezzasalma, et al. 2021). The Baja California Peninsula has a unique geological history and high levels of biodiversity and endemism, where about half of the 144 reptile species are endemic (Dolby, et al. 2015). The study of endemic species in the Baja California Peninsula offers an opportunity to evaluate the effects of geographic isolation in the in situ evolution and speciation at the genomic level.

**Fig. 1.**
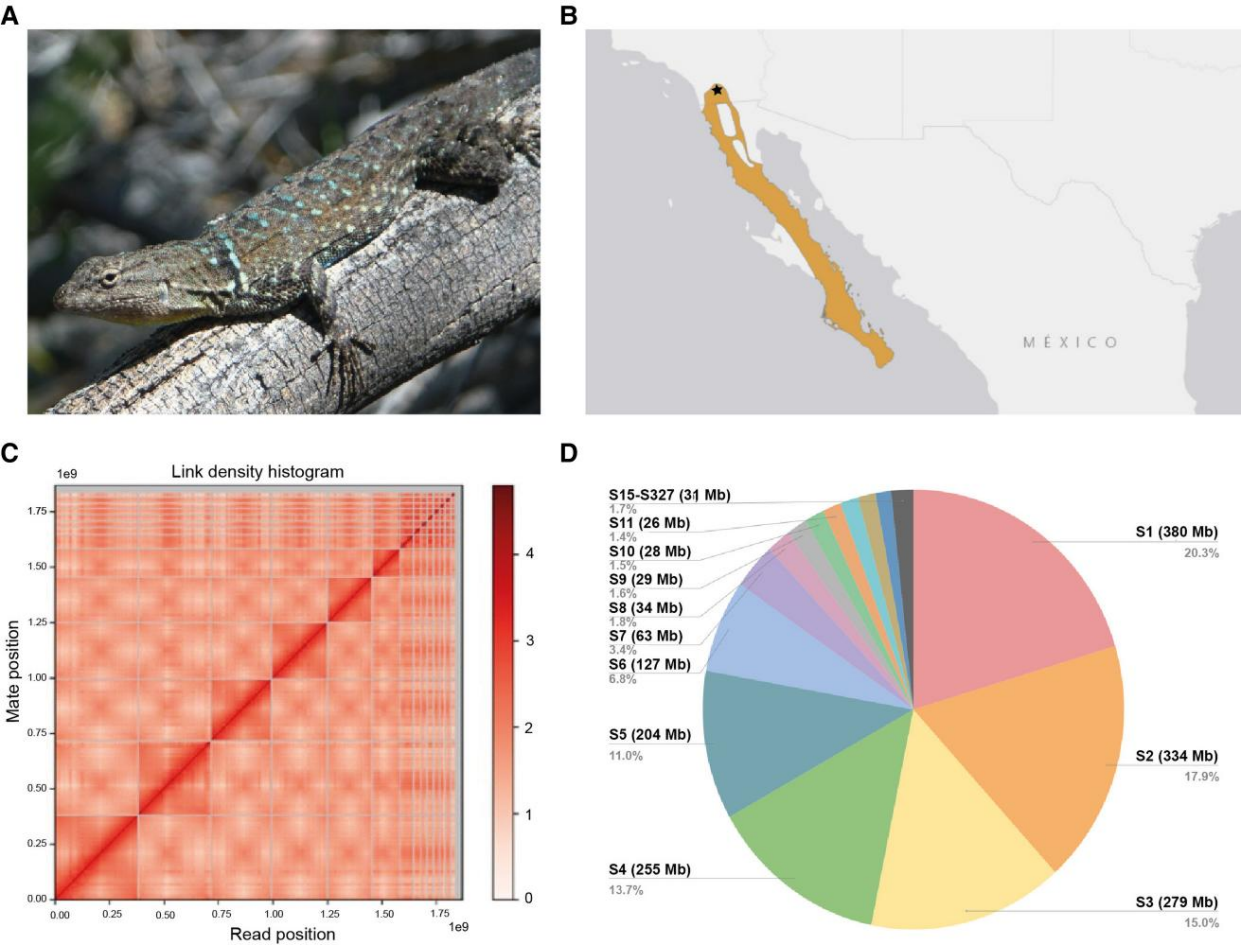
(*a*) Photo of a male *U. nigricaudus* thermoregulating on a tree branch; photo credit: Bruno Granados. (*b*) The current geographical distribution of *U. nigricaudus* in the Baja California Peninsula and southern California, adapted from https://www.iucnredlist.org/. The star indicates where the individual that was sequenced was collected. (*c*) Link density histogram following the Cantata Bio® Omni-C® scaffolding technology and HiRise® software. Six large scaffolds and eight small scaffolds can be observed. (*d*) Pie chart showing the lengths and size percentages with respect to the total sequence for the 14 largest scaffolds (S1–S14). Scaffolds 15–327 (S15–S237; 1.5% of the genome) were combined in the last slice.

*Urosaurus nigricaudus* (NCBI: txid43649) is a lizard species endemic to the Baja California Peninsula in Mexico (fig. 1*a* and *b*). The species’ range extends from southern California, USA, through the Baja California Peninsula and neighboring islands (Aguirre, et al. 1999; Lindell, et al. 2008). This small phrynosomatid lizard, measuring up to 2 g and a maximum of 50 mm in snout-vent length, is well adapted to arboreal life and is often observed climbing trees such as Palo Verde (*Cercidium floridum*) and Mesquite (*Prosopis palmeri*) (Munguia-Vega, et al. 2013). Additionally, it can be found on various cacti and boulders. This lizard is commonly sighted in urban environments (Munguia-Vega, et al. 2013). Due to its small size and ectothermal nature, *U. nigricaudus* relies heavily on the shade provided by desert vegetation to regulate its body temperature and prevent overheating. The species is known for its limited dispersal, where larger males with bright metallic blue throats are territorial and typically bond in a tree with a single female (Grismer 2002; Munguia-Vega, et al. 2013).

*Urosaurus nigricaudus* is a member of the Iguanian suborder, and it is noteworthy that species within this clade, which encompasses iguanas, anoles, and spiny lizards, among others, exhibit a shared XX/XY chromosome system (Rovatsos, et al. 2014) (except basilisks, which possess a more recently evolved XX/XY system (Acosta, et al. 2019)) believed to have originated around 160 Ma (Marin, et al. 2017). However, our understanding of sex chromosomes in iguanids remains limited. Among the iguanids, *Anolis carolinensis* has been subject to more extensive research. Researchers have uncovered that this lizard exhibits a degenerated Y chromosome containing seven identified genes and a dosage compensation mechanism for its entire X chromosome, which serves to equalize gene expression levels of this chromosome between both sexes (Marin, et al. 2017). In other reptiles, such as the Murray River turtle (*Emydura macquarii*, XX/XY), the pink-tailed worm lizard (*Aprasia parapulchella*, XX/XY), the marbled gecko (*Christinus marmoratus*, ZZ/ZW), and the spiny-tailed monitor lizard (*Varanus acanthurus*, ZZ/ZW), researchers have observed a gene-by-gene dosage compensation mechanism rather than a chromosome-wide one (Zhu, et al. 2022). The *Apalone spinifera* (ZZ/ZW) turtle (Bista, et al. 2021) and the pygmy snake *Sistrurus miliarius* (ZZ/ZW) (Vicoso, et al. 2013) have a partial dosage compensation of their Z chromosome. This is interesting because the Apalone turtle's ZZ/ZW and the *A. carolinensis*'s XX/XY systems evolved from the same ancestral autosome, yet they employ distinct dosage compensation strategies (Rovatsos and Kratochvíl 2021).

This work presents a high-quality chromosome-level reference genome and annotation of the black-tail brush lizard, *Urosaurus nigricaudus* (NCBI: txid43649), assembled from PacBio long reads and HiRise scaffolding. The genome of *U. nigricaudus* has 1.87 Gb distributed in 327 scaffolds, with an N50 of 279 Mb and an L50 of 3, and it is composed of 6 macrochromosomes and 8 microchromosomes with ∼17,902 genes. Approximately 46% of the genome is composed of repeated elements with a large proportion of long interspersed nuclear elements (LINEs). Some chromosomes are more prone to rearrangements, but both macro- and microchromosomes are well conserved across Iguanian lizards. The X chromosome shows dosage compensation, and the effective population size for endemic *U. nigricaudus* is very low compared with other squamates.

## Results

### Sequencing and Assembly

The specimen is a male individual collected in 2020 in southern California (fig. 1*a*), United States, near the border with Tecate, Mexico (32.617283° N, −116.735046° W), at the north end of its range; it was collected under California Department of Fish and Wildlife entity permit #838 issued to R. Fisher. DNA was sequenced using the Pacific Bioscience (PacBio) circular consensus long-read sequencing (30× coverage) and Illumina short reads for proximity ligation libraries (see Materials and Methods).

The final genome assembly comprises 1.87 Gb distributed among 327 scaffolds, with an N50 value of 279 Mb, an L50 value of 3, and a genome assembly BUSCO completeness score of 97.65% for Eukaryota genes (table 1). The genome exhibits a GC% content of 40.83%. The Emboss package (v.6.6.0) (Rice, et al. 2000) was utilized to sort and reorder the scaffolds in descending order based on their length. Subsequently, the scaffolds were renamed, starting with the largest one designated as Scaffold 1 and continuing sequentially until Scaffold 327. Approximately 98.4% of the genome is contained within the 14 largest scaffolds (fig. 1*c* and *d*). These include six macrochromosomes (fig. 1*c* and *d*), which account for 84.7% of the genome and range in size from 334 Mb to 127 Mb, as well as eight microchromosomes, constituting 13.7% of the genome and ranging in size from 63 Mb to 22 Mb. Scaffolds numbered 15 to 327 represent unassigned fragments with high levels of repeats (supplementary table S1, Supplementary Material online), with sizes ranging from 1.6 MB to 2 Kb. Microchromosomes have a greater gene density (1.77E^−05^ genes/base pairs) than macrochromosomes (7.67E^−06^; supplementary table S1, Supplementary Material online). We also found that microchromosomes have higher averaged expression levels compared with macrochromosomes (supplementary fig. 1, Supplementary Material online). Additional statistics about the genome assembly can be found in table 1. This Whole Genome Shotgun project has been deposited at DDBJ/ENA/GenBank under the accession JAOEGL000000000. The version described in this paper is version JAOEGL010000000.

**Table 1:** *U. nigricaudus* Genome Assembly Statistics

*U. nigricaudus* HiRise Assembly	
Total length (bp)	1,870,054,407
Scaffold N50 (bp)	279,651,831
Scaffold L50	3
Largest scaffold (bp)	380,405,096
Number of scaffolds	327
Number of scaffolds > 1kbp	327
Number of gaps	200
Number of Ns per 100 kbp	1.09
Complete BUSCOs (C)	249 (97.65%)
Complete and single-copy BUSCOs (S)	248 (97.25%)
Complete and duplicated BUSCOs (D)	1
Fragmented BUSCOs (F)	4
Missing assembly BUSCOs (M)	2
Total BUSCO groups searched	255

### Repeats

The repetitive content within the *U. nigricaudus* genome was identified using RepeatModeler (v2.0.1) (Chen 2004), and subsequent annotation of these elements was performed with RepeatMasker (v4.1.1) (Chen 2004) using known Tetrapoda element databases. We found that ∼46% of the genome consists of repeat elements (table 2). Among the repeat elements covering 43% of the genome, the most abundant ones are transposable elements (TEs), with LINEs being the most abundant type, constituting 23.8% of the genome (table 2). Notably, within the LINE category, families CR1-L3 and L2 are the most prevalent, accounting for 15.75% of the genome. These findings align with previous reports on members of the Iguanian suborder (supplementary table S2, Supplementary Material online), which highlights the dominance of LINEs within the TE landscape. The genome of *U. nigricaudus* has a low frequency of short interspersed nuclear elements (SINEs) at 3.6% and DNA transposons at 8.4% (table 2) similar to other squamate genomes (supplementary table S2, Supplementary Material online).

**Table 2:** Most Common Repeats Present in the *U. nigricaudus* Genome

Transposable elements (43.31%)	LINEs	23.85%	L2/CR1-L3/Rex	15.75%
			R2/R4/Ne	0.04%
			SLRTE/BovB	3.21%
			L1/CIN4	1.76%
	SINEs	3.63%		
	LTR elements	3.66%	BEL/Pao	0.01%
			Ty1/Copia	0.67%
			Gypsy/DIRS1	1.43%
			Retroviral	0.94%
	DNA transposons	8.45%	hobo-Activator	2.85%
			Tc1-IS630-Pogo	2.03%
			PiggyBac	0.04%
			Tourist/Harbinger	3.18%
	Rolling circles	0.05%		
	Unclassified	3.72%		
Simple sequence repeats (2.11%)	Small RNA	0.12%		
	Satellites	0.11%		
	Simple repeats	1.65%		
	Low complexity	0.63%		

Note.—Percentages are relative to the total genome sequence.

**Fig. 2.**
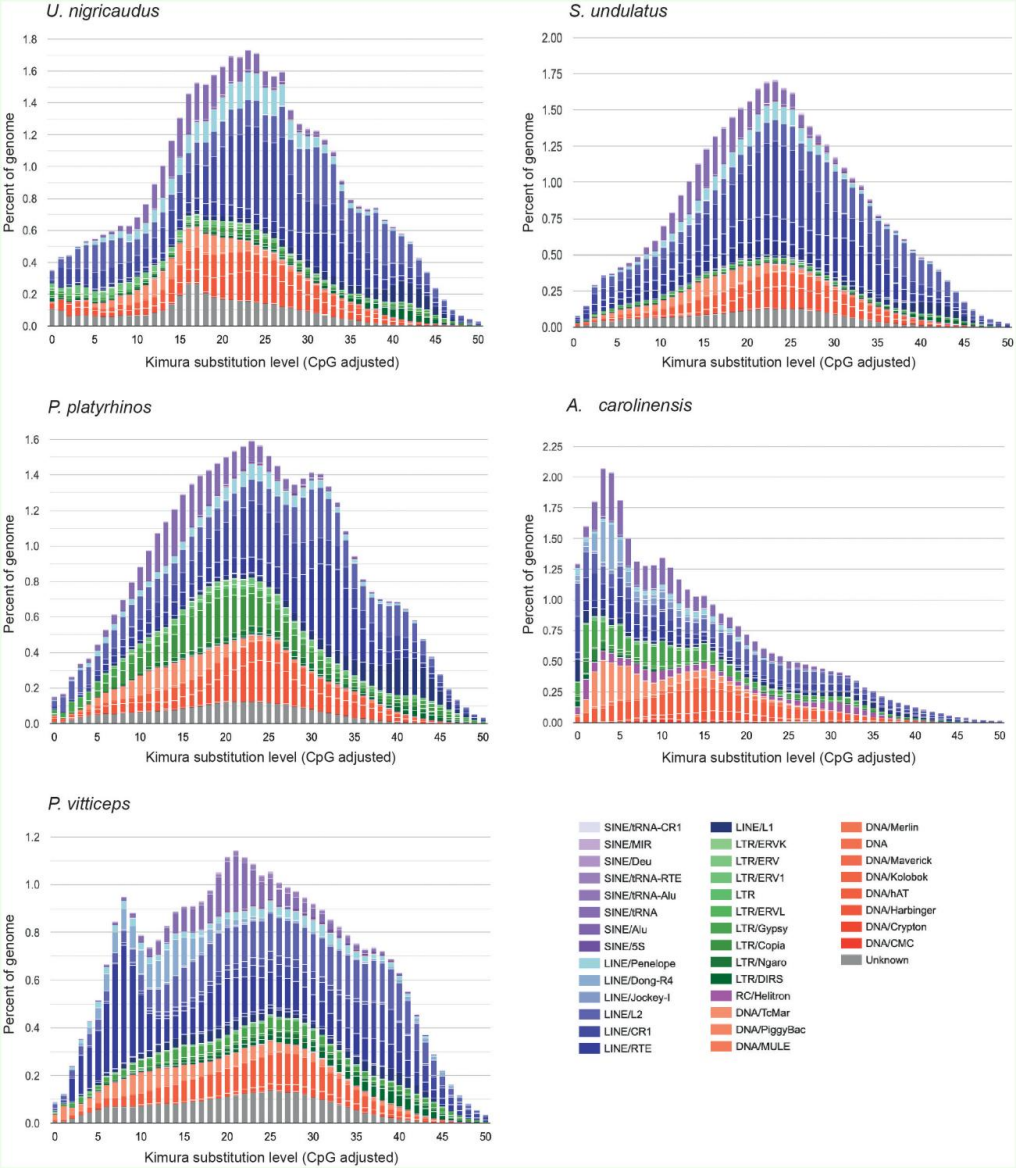
Percentage of the genome (*y* axis) for each TE category with respect to the Kimura distances (*x* axis) in the genomes of *U. nigricaudus*, *S. undulatus*, *P. platyrhinos*, *A. carolinensis*, and *P. vitticeps*. The distribution is based on the Kimura distances (K2) from a consensus sequence. Copies that cluster near K2 = 0 exhibit lesser divergence from the consensus sequence, indicating potential recent activity. In contrast, copies clustering above 10% of divergence from the consensus sequence (K2 > 10) are considered old copies.

We calculated the repeat divergence using the genomes of three lizards within the family Phrynosomatidae (*U. nigricaudus*, *Sceloporus undulatus* (Westfall, et al. 2021), and *Phrynosoma platyrhinos* (Koochekian, et al. 2022)), as well as the genome of two members of the suborder Iguania *A. carolinensis* (Alfoldi, et al. 2011) and *Pogona vitticep*s (Georges, et al. 2015) (fig. 2). We observed a similar distribution in the divergence of repeated elements in the three phrynosomatid lizards (fig. 2), that is, the majority of repeats exhibit ancient activity (K2 > 10), with a prominent peak between 20 and 30 K2. We found that *P. vitticeps* displayed an additional peak representing more recent TE activity between 5 and 10 K2 (fig. 2). A comparison with the *A. carolinensis* genome revealed that this lizard harbors younger repeats (fig. 2), which suggests that TEs have recently been active or are still active in *A. carolinensis*, whereas in *U. nigricaudus* and the other phrynosomatid lizards, purifying selection may not be acting to eliminate repeats (Tollis and Boissinot 2011). The percentage of repeats in the 327 scaffolds can be found in supplementary table S1, Supplementary Material online.

### Gene Annotation

The *U. nigricaudus* genome was annotated using the gene prediction software MAKER v3.01.04 (Cantarel, et al. 2008; Campbell, et al. 2014). MAKER executed 1 round of mapping and 2 rounds of ab initio annotation, identifying a total of 17,902 protein-coding genes. Among these, 17,612 genes (98.38%) exhibited homology with genes from *A. carolinensis*, *S. undulatus*, and *P. platyrhinos*, as determined by BlastP (Altschul, et al. 1990) comparisons. BUSCO analyses (Seppey, et al. 2019) on the annotation, employing the eukaryota_odb10 database, retrieved 84.7% of complete genes (complete, 84.7% [single-copy, 83.9%; duplicated, 0.8%]; fragmented, 10.2%; missing, 5.1%; *n* = 255). Notably, the number of annotated genes in *U. nigricaudus* is comparable with the initial annotations of *A. carolinensis*, *Anolis sagrei*, *S. undulatus*, and *P. platyrhinos*, which reported gene counts of 17,472 (Alfoldi, et al. 2011), 20,033 (Geneva, et al. 2022), 21,050 (Westfall, et al. 2021), and 20,764 (Koochekian, et al. 2022), respectively. Interestingly, 99% of the annotated genes are localized within the first 14 scaffolds, with macrochromosomes harboring 72.5% of the genes and the 8 microchromosomes containing 26.7% (supplementary fig. 2 and tables S1 and S3, Supplementary Material online).

### Synteny Conservation with Other Assemblies

To investigate the genomic synteny of *U. nigricaudus*, we conducted comparative analyses with other reptile genomes that have been assembled at the chromosomal level. Specifically, we compared *U. nigricaudus* against four Iguanian species (*S. undulatus* (Westfall, et al. 2021), *P. platyrhinos* (Koochekian, et al. 2022), *A. carolinensis* (Alfoldi, et al. 2011), *A. sagrei* (Geneva, et al. 2022)), two snake species (*Thamnophis elegans*, https://www.ncbi.nlm.nih.gov/assembly/GCA_009769695.1/, and *Naja naja* (Suryamohan, et al. 2020)), and the common wall lizard *Podarcis muralis* (Lacertidae family; https://www.ncbi.nlm.nih.gov/assembly/GCF_004329235.1/). In this first analysis, we worked with protein-coding genes shared across genomes.

**Fig. 3.**
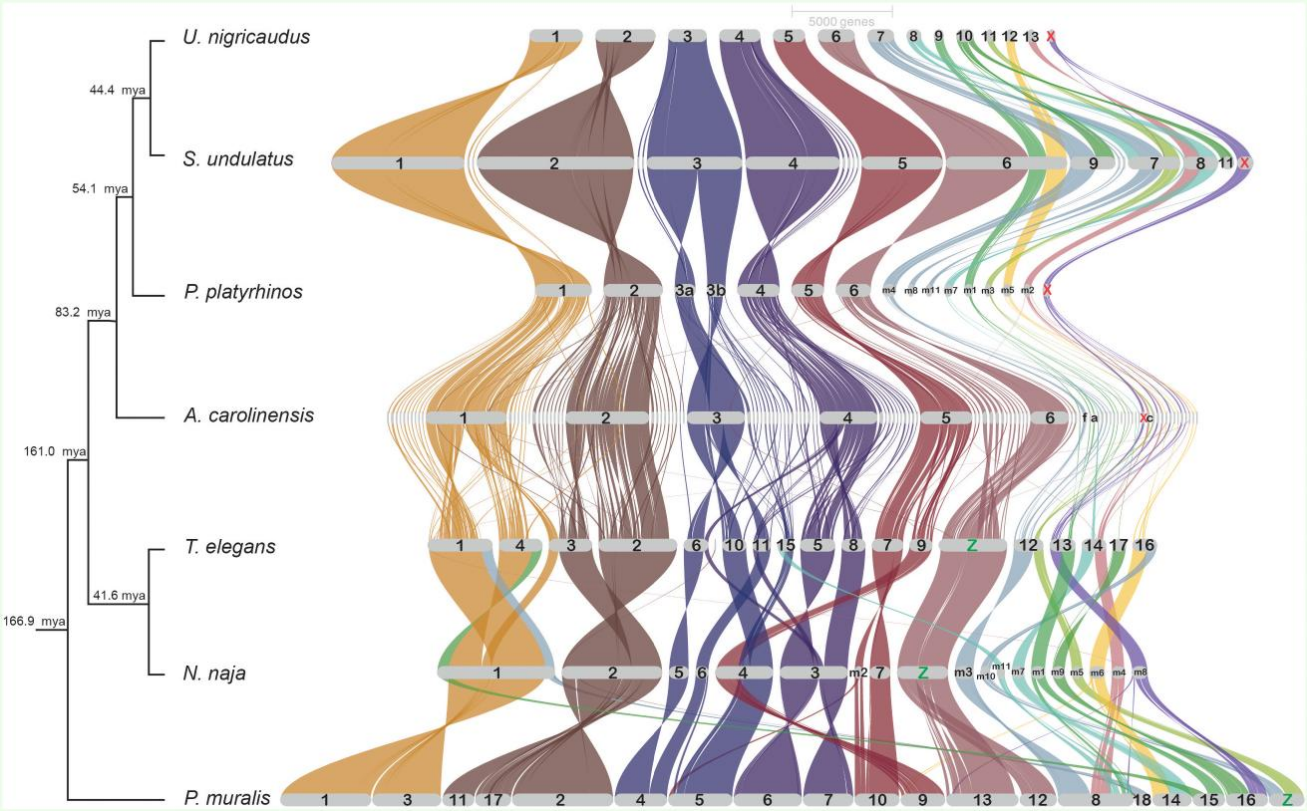
Synteny plot obtained using GENESPACE (Lovell, et al. 2022) of the *U. nigricaudus* genome against chromosome-level assemblies in the Iguania group and more distant species. The color of the linking traits matches orthologous positions between *U. nigricaudus* and the other genomes. Colors are given by the different chromosomes in the *U. nigricaudus* genome. The X chromosome is indicated in place of the scaffold number in all genomes sharing the same XX/XY system: S14 in *U. nigricaudus*, chromosome 10 in *S. undulatus*, microchromosome 9 in *P. platyrhinos*, and chromosome b in *A. carolinensis*. The tree illustrates the divergence between species in millions of years ago (Ma). Divergence estimates were taken from http://timetree.temple.edu/.

Comparing the *U. nigricaudus* genome with other Iguanian species (*P. platyrhinos*, *S. undulatus*, and *A. carolinesis*), we observed a high degree of synteny among the six macrochromosomes (fig. 3, supplementary fig. 3, Supplementary Material online). However, in the microchromosomes, we identified a few rearrangements, such as in S7 and S8. Notably, S14 in *U. nigricaudus* displayed similarity to the already known X chromosome in *S. undulatus*, *P. platyrhinos*, and *A. carolinensis* (supplementary fig. 4, Supplementary Material online) (Alfoldi, et al. 2011; Westfall, et al. 2021; Geneva, et al. 2022; Koochekian, et al. 2022). Expanding the comparative analysis to more distantly related species (*P. muralis* and two snakes), we observed multiple rearrangements in both macro- and microchromosomes (fig. 3, supplementary fig. 5, Supplementary Material online). The only chromosome that exhibited conserved synteny was S6 of *U. nigricaudus*, which corresponded to the Z sex chromosome in both snakes *T. elegans* and *N. naja* (fig. 3, supplementary figs. 4 and 5, Supplementary Material online).

In a previous study by Koochekian et al. (Koochekian, et al. 2022), it was suggested that macrochromosomes might be more susceptible to rearrangements than microchromosomes over long evolutionary timescales. However, the method employed in that study relied on small sequence matches (50 bp) using BlastN (Altschul, et al. 1990), which can generate methodological artifacts when comparing species that diverged over 100 Ma. These artifacts may be amplified when analyzing larger chromosomes.

To address this, we adopted a different approach in our study. We used windows of 10 Kb to map the different chromosomes of *U. nigricaudus* against the other genomes. We quantified the number of significant matches with the chromosomes of species in Iguania (*P. platyrhinos*, *S. undulatus*, *A. carolinensis*, and *A. sagrei*) (supplementary fig. 6*a*, Supplementary Material online) or to more distant species (*N. naja* and *P. muralis*) (supplementary fig. 6*b*, Supplementary Material online). Subsequently, we calculated a “synteny Indei” by dividing the number of matches to the chromosome with the highest count by the total number of matches. This index provided a measure of the preferential matching of a given scaffold to a specific chromosome. A synteny index close to 1 indicates a strong association with a particular chromosome, whereas a decrease in the index value suggests matches with other chromosomes.

Our findings revealed an important conservation of the synteny across Iguania species (supplementary fig. 6*a*, Supplementary Material online). This conservation extends even when analyzing two distantly related squamates (supplementary fig. 6*b*, Supplementary Material online). However, we observed that certain microchromosomes, specifically *U. nigricaudus* S7, S8, S10, and S14, exhibited a higher susceptibility to synteny breaks. Notably, S7 and S14 exhibited a repeat content of over 50%, which could potentially facilitate chromosomal rearrangements. Interestingly, S14 corresponds to the X chromosome in *U. nigricaudus* (supplementary fig. 6*a* and *b*, Supplementary Material online, in red), and sex chromosomes are known to undergo rearrangements that can drive their evolution (Vicoso and Charlesworth 2006). At a larger evolutionary scale (161–167 Ma), some macrochromosomes such as S2 and S4 also displayed synteny breaks, surpassing some microchromosomes. Therefore, it is not universally applicable to assume that a specific class of chromosomes (macro or micro) has a faster evolutionary rate. At least within Iguanians, some microchromosomes (including the X) seem to be evolving more dynamically.

### Sex Chromosome Sequences

With the exception of the Corythophanidae family, all Iguania families share the same XX/XY chromosomes (Altmanova, et al. 2018). Given that the genome assembly was derived from a male individual, we anticipated the presence of scaffolds corresponding to the X and Y chromosomes. To identify these scaffolds, we calculated the coverage in windows of 100 Kb and 1 Kb for macrochromosomes and microchromosomes, respectively.

**Fig. 4.**
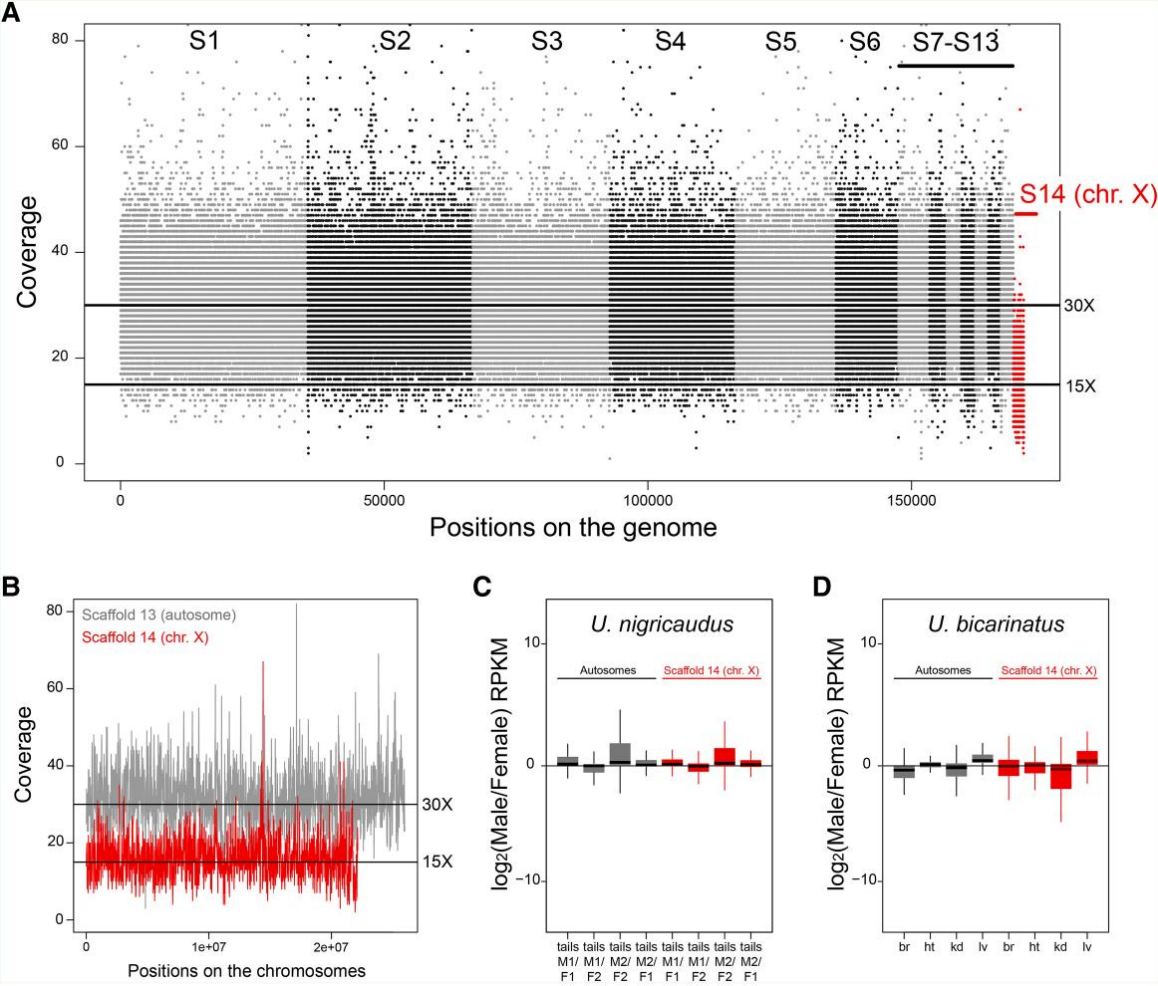
(*a*) Read coverage for scaffolds S1–S14. Each dot represents the average coverage in 100 Kb windows. Scaffolds S1–S13 show an average coverage of 30×. Scaffold S14 (in red), the X chromosome, has half of the coverage (15×). (*b*) Detailed coverage of an autosome (S13) and the X chromosome (S14) using 1 Kb windows. (*c*) Log2 ratio of male against female expression levels of autosomes and the X chromosome in tails. We compared two male samples (M1 and M2) against two female samples (F1 and F2). Transcriptomic data from tails come from *U. nigricaudus*. (*d*) Log2 ratio of male against female expression levels of autosomes and the X chromosome in brain (br), heart (ht), kidney (kd), and liver (lv). Transcriptomic data from somatic tissues come from a related species, *U. bicarinatus* (divergence time: 36 Ma; http://timetree.temple.edu/).

Our analysis revealed that scaffolds S1 to S13 displayed an average coverage of 30× (fig. 4*a*), whereas S14 exhibited half of that coverage (15×) (fig. 4*a*), indicating that this chromosome is present in a single copy in males. Synteny analyses further supported the identification of S14 as the X chromosome, as it exhibited matching patterns with the X chromosome of other closely related lizards (fig. 3). This sex chromosome encompasses 344 genes and is comprised of 50.2% of repeats. Notably, S14 maintained a consistent 15× coverage throughout its sequence (fig. 4*b*), suggesting the absence of a pseudoautosomal region from our assembly.

To investigate whether *Urosaurus* species could exhibit a dosage compensation mechanism for their X chromosome where the single X of males has the same expression levels as two X chromosomes in females, we leveraged transcriptomic data from tails for males and females of *U. nigricaudus* and various somatic tissues for both sexes of *Urosaurus bicarinatus* (a related species with a divergence time of 36 million years; https://timetree.org/). We mapped the RNA-seq reads to our reference genome using HISAT2 (v2.1.0; parameters: -q --threads 16 -a -N 1 -L 18 -i S,1,0.50 -D 20 -R 3 --pen-noncansplice 15 --mp 1,0) (Kim, et al. 2015) and compared expression values (RPKM) between male and female samples. To facilitate the analysis, we divided the autosomes and the X chromosome in windows of 10 Kb and 1 Kb, respectively, and calculated the expression level of these windows using deepTools (Ramirez, et al. 2014). Our analysis revealed similar male-to-female expression ratios for both autosomes and the X chromosome (fig. 4*c* and *d*; supplementary fig. 7, Supplementary Material online), as well as a similar expression between the X chromosome and autosomes (supplementary fig. 1, Supplementary Material online), indicative of a full chromosome-wide dosage compensation mechanism for the X chromosome in *U. nigricaudus* and also very likely in *U. bicarinatus*. Given that a similar mechanism is present in *A. carolinensis* (Marin, et al. 2017), we hypothesized that the dosage compensation system that balances the expression levels of X chromosomes in Iguanian species could be ancestral and more than 100 million years old.

Regarding the Y chromosome, we initially considered scaffolds S15 and S16 as potential Y-linked candidates, as they exhibited a 15× coverage (supplementary fig. 8, Supplementary Material online), contained a relatively small number of genes (6 and 2, respectively; supplementary table S1, Supplementary Material online), and were predominantly composed of repeated sequences (81.7% and 87.3%, respectively; supplementary table S1, Supplementary Material online). These characteristics are in agreement with what we know about Y chromosomes in Iguania (Marin, et al. 2017). However, our analysis of male-to-female expression ratios failed to reveal any male-specific expression (supplementary fig. 8, Supplementary Material online).

### Effective Population Size

We conducted pairwise sequential Markovian coalescent (PSMC) (Li and Durbin 2011) analyses on the *U. nigricaudus* genome and other related species within the Iguania group. Considering the effects of restricted distribution and the effects of glacial cycles (i.e., bottlenecks, isolation/reconnectivity) on a long and narrow peninsula, we anticipated a relatively small effective population size for *U. nigricaudus*. In contrast, we knew that the *P. vitticeps* genome had revealed a large number of polymorphisms and a correspondingly large effective population size (Georges, et al. 2015). Given the similar size and location of the geographical distributions of *A. carolinensis*, *S. undulatus*, and *P. platyrhinos*, we expected comparable population sizes among these species.

**Fig. 5.**
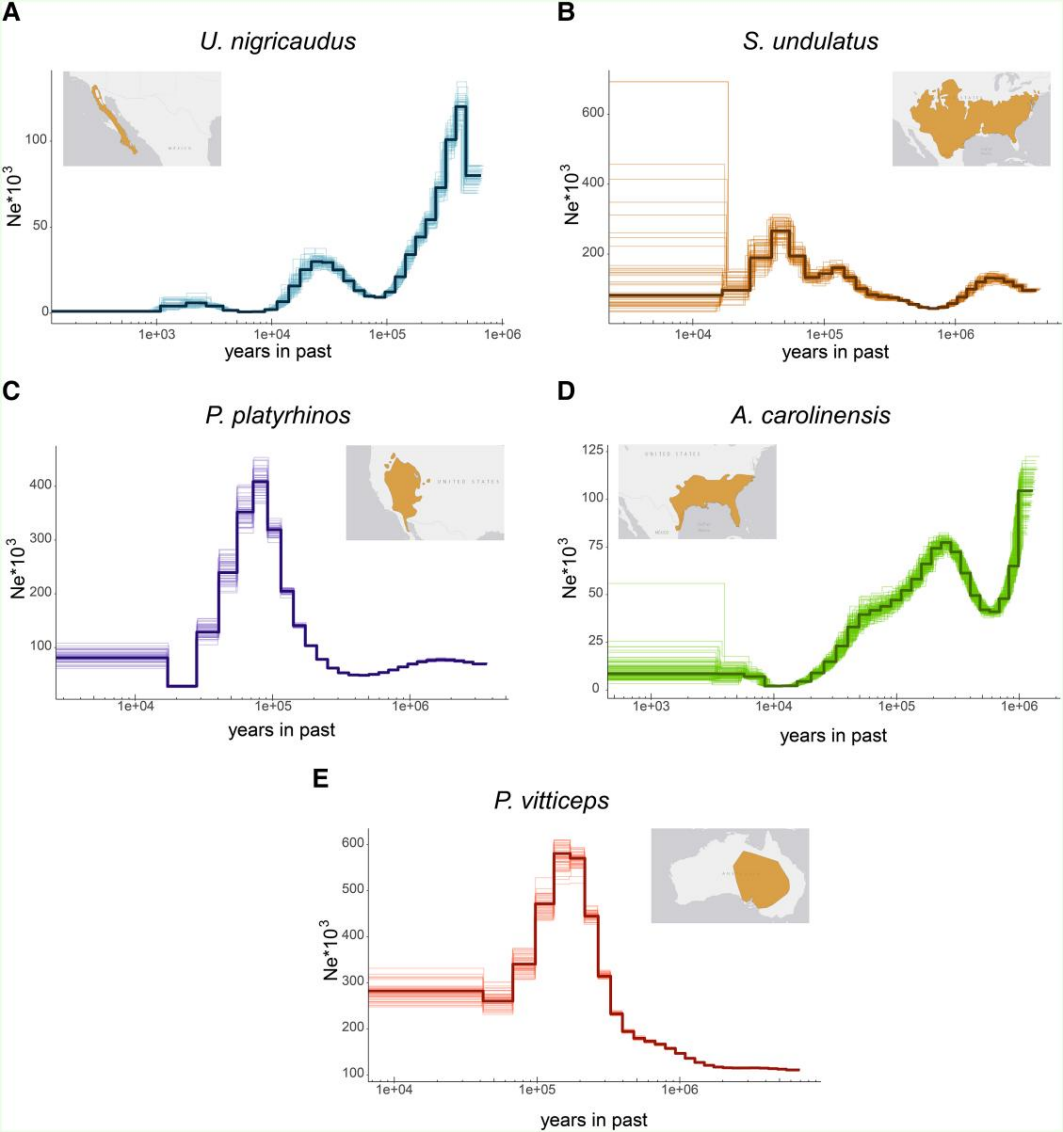
Changes over time in the population size of *U. nigricaudus*, *S. undulatus*, *P. platyrhinos*, *A. carolinensis*, and *P. vitticeps* obtained from PSMC analysis of each reference genome. Maps show the current geographical distribution of the species. Maps were adapted from https://www.iucnredlist.org/.

Our PSMC results aligned with our expectations. *U. nigricaudus*, approaching Ne = 0, exhibited the smallest estimated current population size among the five species. A sharp decline in population size likely began ∼500,000 years ago with an increase between 100,000 and 25,000 years ago and a continuous decline since then (fig. 5*a*). Notably, *A. carolinensis* displayed a similar pattern to *U. nigricaudus*, with an extremely low estimated current effective population size, though it has less of a tree-obligate habitat use pattern than *U. nigricaudus* (fig. 5*d*). The other three species indicated their peak effective population sizes around 100,000 years ago, followed by a rapid decline in *S. undulatus* (fig. 5*b*) and *P. platyrhinos* (fig. 5*c*). In contrast, *P. vitticeps* (fig. 5*e*) still maintains a large estimated current effective population size.

## Discussion

The *U. nigricaudus* genome assembly presented in this work demonstrates exceptional contiguity, with only 327 scaffolds, a scaffold N50 of over 279 Mb, and ∼98.4% of the genome contained within the 14 largest scaffolds. This assembly ranks among the most contiguous squamate genomes available in the NCBI database. Notably, it represents the first genome assembly for the *Urosaurus* genus and the fourth assembly at a chromosome level within the Phrynosomatidae family in the NCBI database. Being an endemic lizard, the *U. nigricaudus* genome could shed light on the evolutionary role of restricted dispersal, small population size, and genetic drift due to isolation and the coevolution with vegetation during the expansion and contraction of desert habitats during glacial and interglacial periods (Dolby, et al. 2015), which could explain the oscillatory changes observed in historical effective size from the PSMC analysis. Also, due to its small size and lack of burrowing behavior which translates into a high risk of overheating, the *Urosaurus* genome could inform about the physiological limits on activity time, adaptation capacity, and extinction risk of ectotherms to global warming and deforestation.

We identified the 14 largest scaffolds as potential chromosomes of *U. nigricaudus*, suggesting a potential chromosomal count of 2*n* = 28 (six pairs of macrochromosomes and eight pairs of microchromosomes). The ancestral karyotype for iguanids and spiny lizards probably exhibited 6 pairs of macrochromosomes but 11 pairs of microchromosomes (2*n* = 34) (Leache and Sites 2009). The karyotype of two *Urosaurus* species has been described (*Urosaurus graciosus* (Gorman 1973) and *Urosaurus ornatus* (Porter et al. 1994 )). In both cases, the karyotype is also 2*n* = 34; 6 macrochromosomes and 11 microchromosomes. According to this data, the number of macrochromosomes in our assembly is correct (*n* = 6), but it appears that we are currently missing three microchromosomes that may be in the unassembled scaffolds or have been fused with other chromosomes. To validate the exact number of chromosomes in *U. nigricaudus*, especially at the microchromosome level, a karyotype of the species analysis is necessary.

We also identified scaffold 14 as the X chromosome in this species exhibiting a perfect chromosome-wide dosage compensation mechanism that seems to be shared with *Anolis* (Marin, et al. 2017). The current assembly of the X chromosome has no pseudoautosomal region. We found that this result is most likely due to a break in the assembly. We would require female genomic data to identify a potential scaffold showing the pseudoautosomal boundary. At present, it seems more likely that the Y chromosome is absent from our assembly as we failed to identify other scaffolds with half of the genomic coverage (i, e., one copy in males), with male-specific expression or showing orthology with Y-linked genes reported for *A. carolinensis*. We are planning a dedicated approach to work on a Y-specific assembly. Moreover, scaffolds S15 and S16 may represent unassembled segments of the X chromosome.

The annotation process yielded a protein-coding gene count (17,902) comparable with the first annotation of *A. carolinensis* (17,472), demonstrating the effectiveness of utilizing a transcriptome from a closely related species when genomic data are limited. However, the BUSCO analysis for the annotated transcripts indicates that further improvement in the annotation could be achieved, probably using a larger transcriptomic data set. Furthermore, the repeat landscape of *U. nigricaudus* exhibits similarities to other Iguanian lizards, whereas the repeat divergence pattern aligns with that observed in other phrynosomatid lizards, where the majority of TEs were active during ancient times.

Microchromosomes in vertebrates exhibit distinctive characteristics, including a notably high gene density, elevated recombination rates, an elevated GC content, and an abundance of CpG islands (Srikulnath, et al. 2021). The study of these attributes in the microchromosomes of *U. nigricaudus* highlights their conservation. This conservation, particularly in terms of higher GC content, is likely linked to the increased gene expression levels observed in microchromosomes compared with macrochromosomes. Synteny analysis revealed intriguing patterns with most of the macro- and microchromosomes showing a high degree of conservation across reptiles with a few exceptions of microchromosomes that have been more prone to synteny disruptions. This suggests potential microchromosome fusions and/or fissions that occurred during the evolution of these species. It is noteworthy that many microchromosomes have not undergone significant rearrangements. This observation aligns with a recent change in notion, indicating that microchromosomes should be viewed as fundamental building blocks of amniote chromosomes, rather than as aberrant genetic elements (Waters, et al. 2021).

The significance of high-quality and contiguous genome assemblies cannot be overstated, as they serve as crucial references for a wide range of genomic analyses, including investigations into biology, physiology, demography, ecology, and evolution. As *U. nigricaudus* is endemic to the Baja California Peninsula, this genome assembly will provide valuable insights into the species’ evolution and its population genomic characteristics within the unique ecological and geological context of the peninsula. Furthermore, alongside other reptile reference genomes, we anticipate that this squamate genome assembly will contribute significantly to the evolutionary analysis of this diverse group.

## Materials and Methods

### DNA Samples and Sequencing

The type specimen is a male individual collected in 2020 in southern California (fig. 1*a*), United States, near the border with Tecate, Mexico (32.617283° N, −116.735046° W), at the north end of its range; it was collected under California Department of Fish and Wildlife entity permit #838 issued to R. Fisher. The specimen was transported to Tempe, Arizona, and housed for 1 week prior to being euthanized in laboratory conditions using ∼100 mg/km sodium pentobarbital solution via intracoelomic injection. Skeletal muscle, liver, and blood tissues were collected and flash-frozen in liquid nitrogen and then stored at −80 °C. Procedures were approved by the Institutional Animal Care and Use Committee (IACUC) at Arizona State University under protocol #20-1737R RFC 2 issued to G.A.D. Tissues were shipped to Cantata Bio LLC for high molecular weight DNA extraction and library preparation. DNA samples were quantified using Qubit 2.0 Fluorometer (Life Technologies, Carlsbad, CA, USA). The PacBio SMRTbell library (∼20 kb) for PacBio Sequel was constructed using SMRTbell Express Template Prep Kit 2.0 (PacBio, Menlo Park, CA, USA) using the manufacturer-recommended protocol. The library was bound to polymerase using the Sequel II Binding Kit 2.0 (PacBio) and loaded onto PacBio Sequel II. DNA was sequenced using the PacBio Sequel II 8M SMRT cell circular consensus long-read sequencing (CCS reads; 30× coverage) and Illumina short reads for the Omni-C® chromatin proximity ligation libraries.

### Assembly

Contigs were developed from 58 Gbp of PacBio data by the hifiasm® software pipeline, and scaffolding was performed using the Omni-C® HiRise® pipeline by Cantata Bio (https://cantatabio.com). Fifty-eight gigabase pairs of PacBio CCS reads were used as an input to Hifiasm1 v0.15.4-r347 with default parameters. BlastN (Altschul, et al. 1990) results of the Hifiasm output assembly against the nt database were used as input for blobtools2 v1.1.1, and scaffolds identified as possible contamination were removed from the assembly. Finally, we removed haplotigs and contig overlaps. Next, for each Omni-C library, chromatin was fixed in place with formaldehyde in the nucleus and then extracted. Fixed chromatin was digested with DNAse I, and chromatin ends were repaired and ligated to a biotinylated bridge adapter followed by proximity ligation of adapter containing ends. After proximity ligation, crosslinks were reversed and the DNA was purified. Purified DNA was treated to remove biotin that was not internal to ligated fragments. Sequencing libraries were generated using NEBNext Ultra enzymes and Illumina-compatible adapters. Biotin-containing fragments were isolated using streptavidin beads before PCR enrichment of each library. The library was sequenced on an Illumina HiSeqX platform to produce ∼30× sequence coverage. HiRise used MQ > 50 reads for scaffolding. The input de novo assembly and Omni-C library reads were used as input data for HiRise, a software pipeline designed specifically for using proximity ligation data to scaffold genome assemblies. Omni-C library sequences were aligned to the draft input assembly using Burrows–Wheeler Aligner (BWA) (Li and Durbin 2009). The separations of Omni-C read pairs mapped within draft scaffolds were analyzed by HiRise to produce a likelihood model for genomic distance between read pairs, and the model was used to identify and break putative misjoins, to score prospective joins, and make joins above a threshold. This Whole Genome Shotgun project has been deposited at DDBJ/ENA/GenBank under the accession JAOEGL000000000.

### Repeats

The repetitive content within the *U. nigricaudus* genome was identified using RepeatModeler (v2.0.1) (Chen 2004), and subsequent annotation of these elements was performed with RepeatMasker (v4.1.1) (Chen 2004) using known Tetrapoda element databases. To obtain a summary of repeat composition, ProcessRepeats, a tool within RepeatMasker, was employed. To assess the evolutionary divergence of repeated elements within the *U. nigricaudus* genome, we employed the calcDivergenceFromAlign.pl tool in RepeatMasker (Chen 2004). This tool calculates Kimura’s divergence based on alignments generated by RepeatMasker.

### Gene Annotation

The *U. nigricaudus* genome was annotated using the gene prediction software MAKER v3.01.04 (Cantarel, et al. 2008; Campbell, et al. 2014). MAKER used known proteins, EST evidence, and an organism-specific repeat library to train ab initio prediction programs Augustus (Keller, et al. 2011) and SNAP (Johnson, et al. 2008). For this analysis, we provided MAKER with complete protein sets from *A. carolinensis* (AnoCar2.0) and *S. undulatus* (SceUnd_v1.1), acquired from the NCBI database. Additionally, transcriptomic data in the form of EST evidence from a closely related species (*U. bicarinatus*) were incorporated, comprising ∼187 million 101-nt-long paired-end reads for brain, heart, kidney, liver, and gonad samples from one male and one female individual. Information pertaining to the identified and annotated repeated elements using RepeatMasker was also included.

### Synteny Conservation with Other Assemblies

We used BWA (Li and Durbin 2009) to search for the nucleotide sequences of annotated genes from *U. nigricaudus* in the genomes of the other species. We specified the parameters as bwa mem -t 15. For each gene, we retained only the best hit. To visualize the synteny relationships, we employed the R language (R Core Team 2021), GENESPACE (v1.3.1) (Lovell, et al. 2022) for the seven genome alignment, and Circos (v.0.69-9) (Krzywinski, et al. 2009) for the paired comparisons between *U. nigricaudus* and the other species.

We divided the *U. nigricaudus* genome into windows of 10 Kb and mapped them against the genomes of other species using BWA (Li and Durbin 2009). We specified the parameters as bwa mem -t 30. For each scaffold in *U. nigricaudus*, we quantified the number of significant matches with the chromosomes of species in Iguania (*P. platyrhinos*, *S. undulatus*, *A. carolinensis*, and *A. sagrei*) or to more distant species (*N. naja* and *P. muralis*). Subsequently, we calculated a “synteny index” by dividing the number of matches to the chromosome with the highest count by the total number of matches. This index provided a measure of the preferential matching of a given scaffold to a specific chromosome. A synteny index close to 1 indicates a strong association with a particular chromosome, whereas a decrease in the index value suggests matches with other chromosomes.

### RNA Samples and Sequencing

Two males and two females of *U. nigricaudus* were captured in Santa Rita (23.3279699° N, −109.7016934° W) Baja California, Mexico. Tails were collected and flash-frozen in liquid nitrogen. Tissues were shipped to Cantata Bio LLC for total RNA extraction and library preparation. Additionally, one male and one female *U. bicarinatus* were captured near El Limón (18.531466° N, −98.9416673° W) in Morelos, Mexico. The specimens were housed for 1 day prior to being euthanized using ∼100 mg/km sodium pentobarbital solution via intracoelomic injection. Brain, heart, kidney, liver, and the gonads were collected and flash-frozen in liquid nitrogen and then stored at −80 °C. RNA was extracted, used to generate strand-specific RNA-seq libraries (using the Illumina TruSeq Stranded mRNA Library protocol), and sequenced on an Illumina NextSeq 500 platform at UNAM's sequencing facility (http://www.uusmb.unam.mx/).

### Sex Chromosome Sequences

We employed a coverage-based approach. Specifically, we mapped the original raw PacBio reads to the assembled genome using BWA (bwa mem -t 30) (Li and Durbin 2009) and calculated the coverage in windows of 1 Kb and 100 Kb using deepTools v.3.3.1 (Ramirez, et al. 2014). Moreover, RNA-seq data were trimmed for adaptors and low-quality positions using trim_galore (v0.6.2) (https://github.com/FelixKrueger/TrimGalore). We then mapped the RNA-seq reads from *U. nigricaudus* (∼132 million 101-nt-long paired-end reads for tail samples from two male and two female individuals) and *U. bicarinatus* (∼187 million 101-nt-long paired-end reads for brain, heart, kidney, and liver samples from one male and one female individuals) to our reference genome using HISAT2 (v2.1.0; parameters: -q --threads 16 -a -N 1 -L 18 -i S,1,0.50 -D 20 -R 3 --pen-noncansplice 15 --mp 1,0) (Kim, et al. 2015) and compared expression values (RPKM) between male and female samples. We obtained >86% and >79% of aligned reads for *U. nigricaudus* and *U. bicarinatus*, respectively. To facilitate the analysis, we divided the autosomes and the X chromosome in windows of 10 Kb and 1 Kb, respectively, and calculated RPKM values of these windows using deepTools (Ramirez, et al. 2014). We used R (R Core Team 2021) to compile genomic read coverage or the RPKM values into dataframes, calculate log2 male/female ratios for the RNA-seq data, and plot the results.

### Effective Population Size

We conducted PSMC (Li and Durbin 2011) analyses on the *U. nigricaudus* genome and other related species within the Iguania group. We first mapped the raw sequencing reads to their corresponding reference genome using BWA (bwa mem -t 20). Raw reads were downloaded from the NCBI database. The number of reads to be mapped was established so we could get ∼30× of coverage. The sorted BAM files were analyzed with samtools mpileup (samtools mpileup -Q 30 -q 30 -u -v -f) followed by bcftools (bcftools call -c) and vcfutils.pl (vcfutils.pl vcf2fq -d 3 -D 30 -Q 30). We then implemented PSMC (psmc -N25 -t15 -r5 -p “4+25*2+4+6”) with 100 bootstraps and plotted the results in intermediate files (psmc_plot.pl -u 1.17e-8 -g 3 -R). The final plotting was obtained using the ggplot2 library (Wickham 2016) in R (R Core Team 2021). For the calculations, we used the average reptile mutation rate of 1.17 × 10^−8^ (Bergeron, et al. 2023) and a generation time of 1.

## Supplementary Material

Supplementary data are available at *Genome Biology and Evolution* online (http://www.gbe.oxfordjournals.org/).

## Supplementary Material

evad210_Supplementary_Data

## Data Availability

The reference genome has been deposited in the DDBJ/ENA/GenBank under the accession JAOEGL000000000. RNA-seq data from *U. nigricaudus* were deposited in the NCBI-SRA under BioProject PRJNA1024475. RNA-seq data from *U. bicarinatus* were deposited in the NCBI-SRA under BioProject PRJNA998397. Data sets and scripts were deposited in GitHub https://github.com/dcortezccg/Urosaurus_nigricaudus_supp_data/tree/main.
