# Supplementary material for: Chromosome-Level Genome Assembly of the Blacktail Brush Lizard, Urosaurus nigricaudus, Reveals Dosage Compensation in an Endemic Lizard
Source: Genome Biol Evol. 2023 Dec 6;15(12):evad210. doi: 10.1093/gbe/evad210 (PMC10699878; doi:10.1093/gbe/evad210)
Supplement: evad210_Supplementary_Data [file evad210_supplementary_data.zip › Davalos-Dehullu.et.al.2023.Supp.tables2-3.and.figures1-8.pdf]

Supplementary table 2. Common repeats in eight species belonging to the Iguanian sub-order.

	Total Repeats	LINES	LTR Elements	SINES	DNA transposons	Satellites	Unclassified	Reference
U. nigricaudus	45.86 %	23.85 %	3.66 %	3.63 %	8.45 %	0.11 %	3.72 %	This study
Phrynosoma platyrhinos	44.45 %	16.90 %	2.22 %	4.72 %	10.80 %	6.9 %	7.72 %	(Koocheki an, et al. 2022)
Phrynosoma cornutum	49.56 %	16.46 %	2.84 %	4.05 %	10.63 %	1.89 %	6.98 %	(Pasquesi, et al. 2018)
Sceloporus poinsettii	45.49 %	16.95 %	2.37 %	2.66 %	7.87 %	1.81 %	6.1 %	
Sceloporus teapensis	45.36 %	19.83 %	3.83 %	4.16 %	11.22 %	1.33 %	6.29 %	
Sceloporus undulatus	41.33 %	14.77 %	1.33 %	3.5 9 %	8.82 %	0.60 %	12.22 %	(Westfall, et al. 2021)
Anolis sagrei	51.69 %	22.85 %	2.16 %	2.90 %	11.35 %	ND	9.96 %	(Geneva, et al. 2022)
Anolis carolinensis	51.54 %	17.66 %	3.67 %	1.4 %	7.93 %	1.74 %	5.7 %	(Pasquesi, et al. 2018)

Supplementary table 3. *U. nigricaudus* annotated features.

Total annotated genes	17,902
Exons	161,822
CDS	156,472
5’ UTR	10,191
3’ UTR	12,502
Average gene length	1850 bp

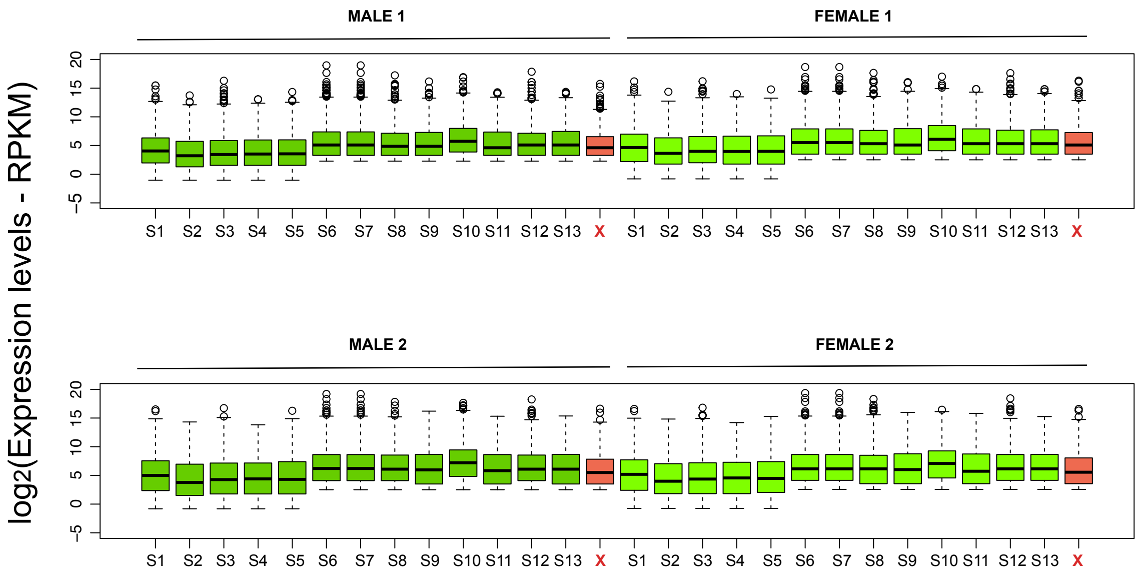

Supplementary figure 1. Boxplots showing the expression levels (RPKM) for 6 macrochromosomes and 8 microchromosomes, including the X chromosome, using data from tails of two males and two females. The X chromosome (red) has similar expression levels as the microchromosomes (S7-S13; in green). Scaffold 6, although considered a macrochromosome, has a more similar expression pattern to the microchromosomes.

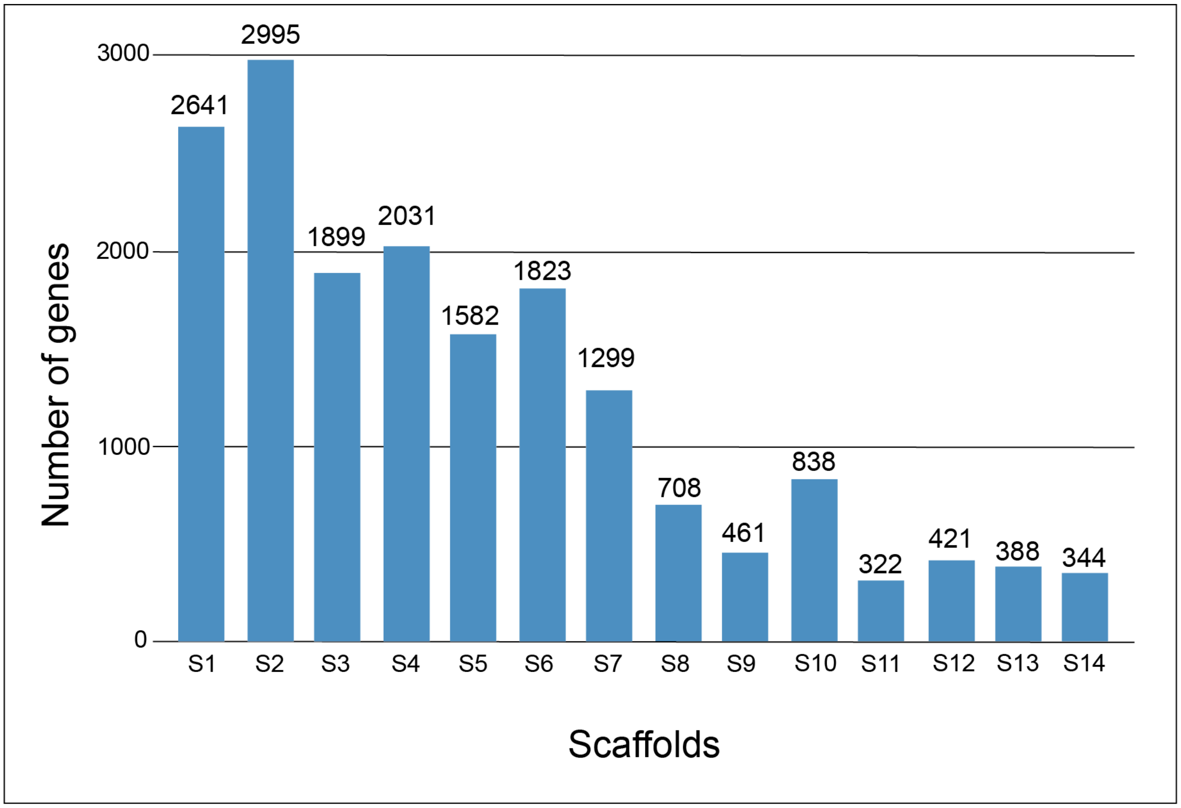

Supplementary figure 2. Number of genes in scaffolds S1-S14 corresponding to 6 macro and 8 microchromosomes.

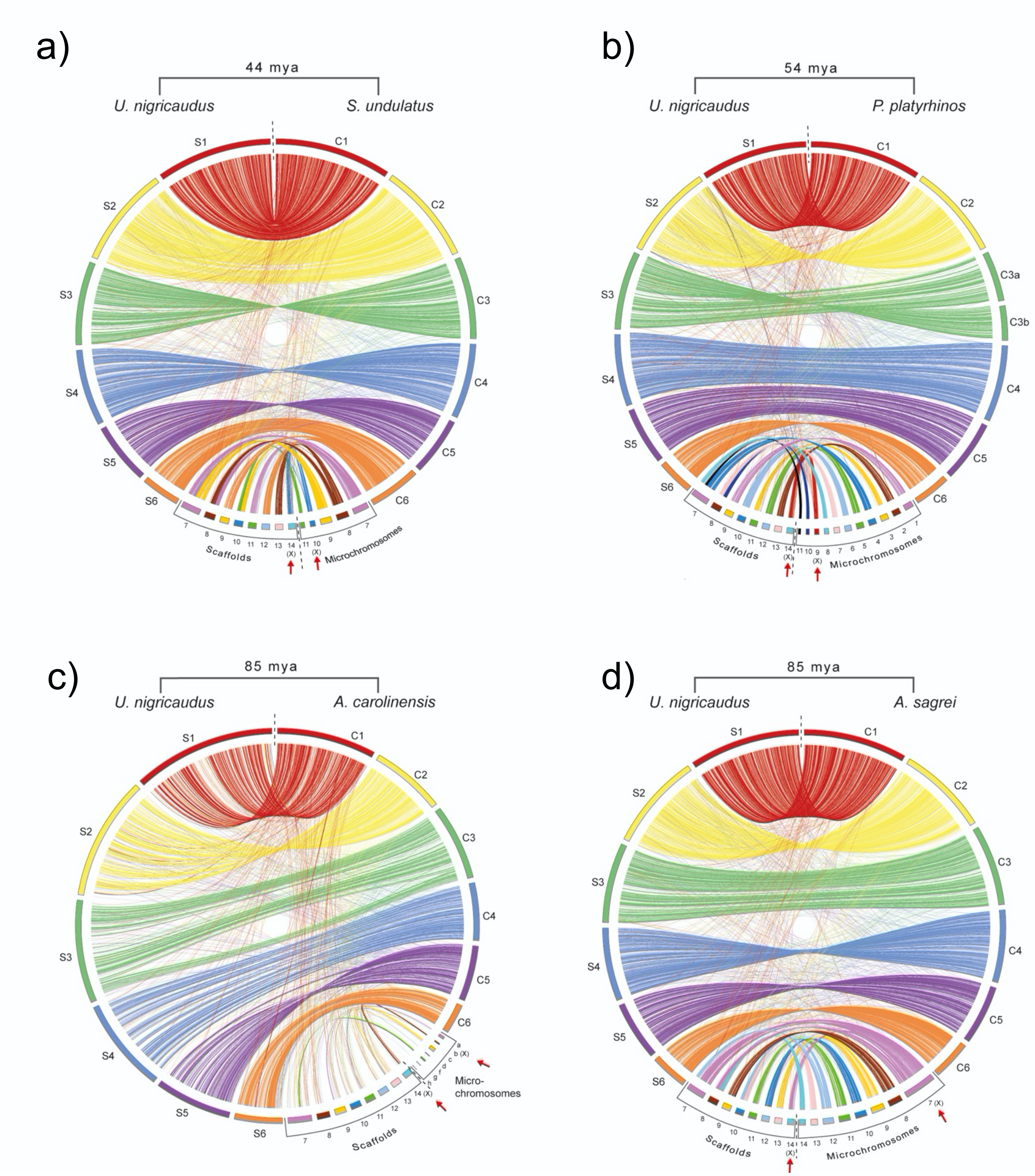

Supplementary figure 3. Synteny plots of the *U. nigricaudus* genome against other individual chromosome-level assemblies in the Iguania group. The color of the linking traits matches orthologous positions between *U. nigricaudus* and the other genomes. Colors are given by the different chromosomes in the *U. nigricaudus* genome. S indicates scaffold whereas C indicates

chromosome. Dotted lines indicate the boundaries of the two genomes being compared. Red arrows signal the relationship between S14 in *U. nigricaudus* with known X chromosomes. The divergence time between species is shown on top of the species names. Divergence estimates were taken from http://timetree.temple.edu/. a) *U. nigricaudus* against *S. undulatus*. b) *U. nigricaudus* against *P. platyrhinos*. c) *U. nigricaudus* against *A. carolinensis*. d) *U. nigricaudus* against *A. sagrei*.

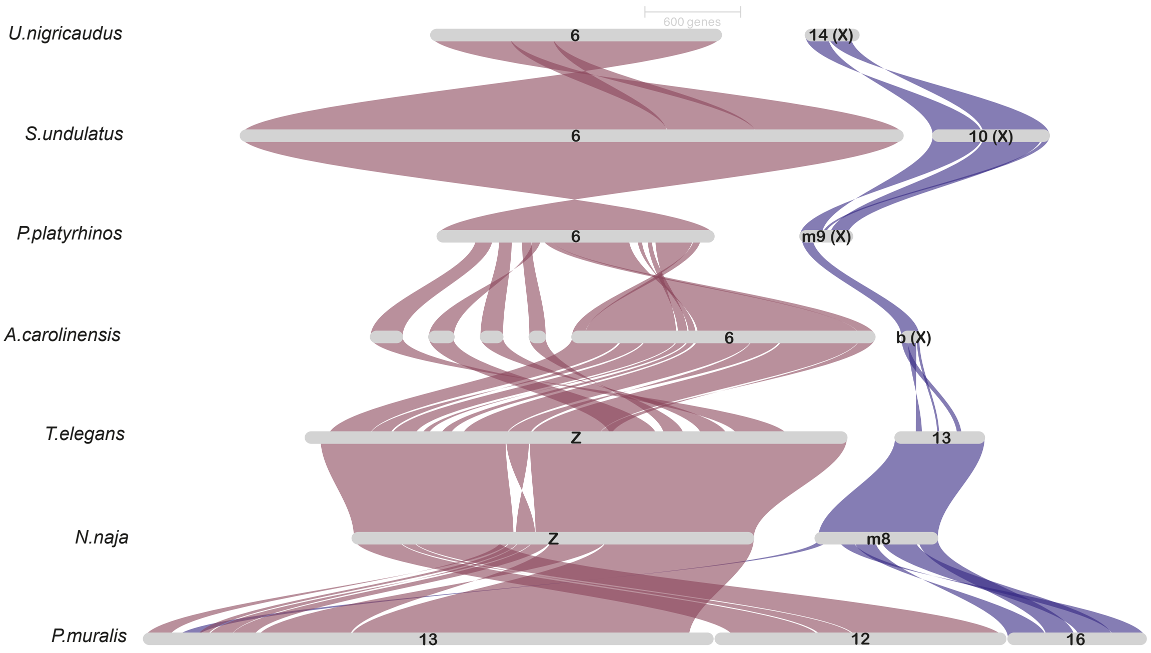

Supplementary figure 4. Synteny plot obtained using genespace (Lovell, et al. 2022) of the *U. nigricaudus* genome against other species for Scaffolds 14 (the X chromosome in iguanian) and Scaffold 6 (the Z chromosome in snakes). The color of the linking traits matches orthologous positions between *U. nigricaudus* and the other genomes. Colors are given by the different chromosomes in the *U. nigricaudus* genome.

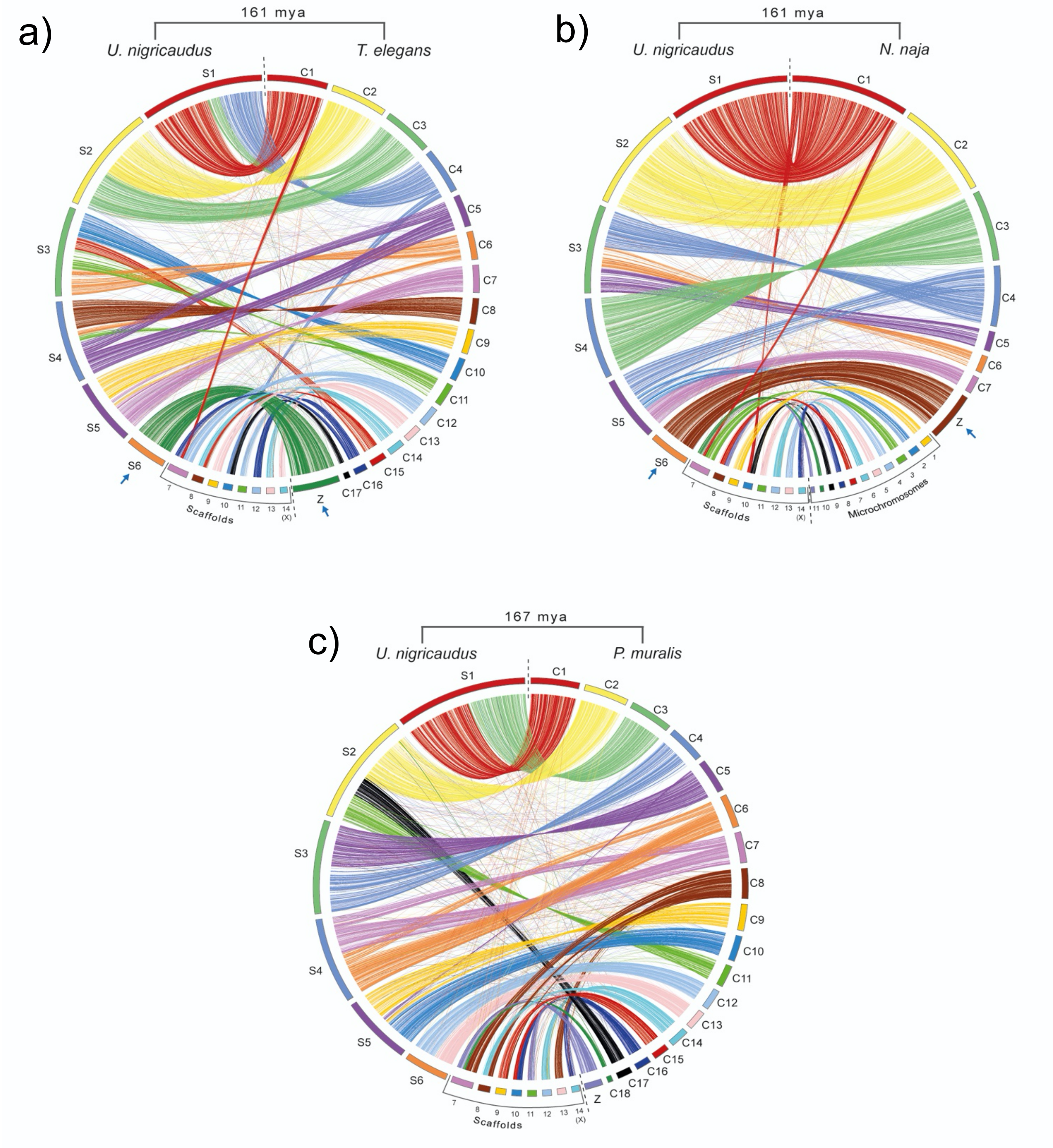

Supplementary figure 5. Synteny plots of the *U. nigricaudus* genome against other individual chromosome-level assemblies from more distantly related reptile species. The color of the linking traits matches orthologous positions between *U. nigricaudus* and the other genomes. Colors are given

by the different chromosomes in the *U. nigricaudus* genome. S indicates scaffold whereas C indicates chromosome. Dotted bars indicate the boundaries of the two genomes that are being compared. Blue arrows signal the relationship between Scaffold 6 in *U. nigricaudus* with known Z chromosomes in snakes. The divergence time between species is shown on top of the species names. Divergence estimates were taken from http://timetree.temple.edu/. a) *U. nigricaudus* against *T. elegans*. b) *U. nigricaudus* against *N. naja*. c) *U. nigricaudus* against *P. muralis*.

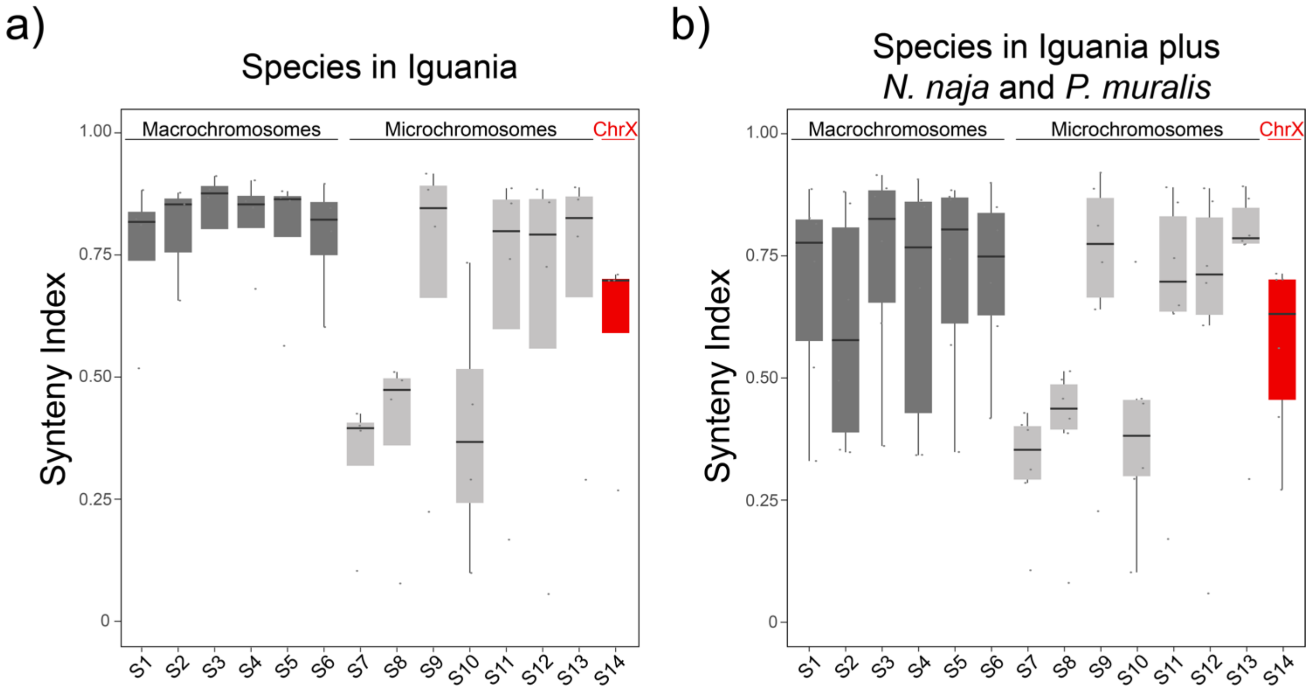

Supplementary figure 6. Boxplots summarizing the values of the synteny index for the six macrochromosomes (in dark grey; S1-S6) and the eight microchromosomes (light grey; S7-S13), including the X chromosome (in red; S14). a) Synteny indexes between species that belong to the Iguania clade: *P. platyrhinos*, *S. undulatus*, *A. carolinenesis,* and *A. sagrei*. b) Synteny indexes across Iguania and two more distant species: the cobra (*N. naja*) and the common wall lizard (*P. muralis*).

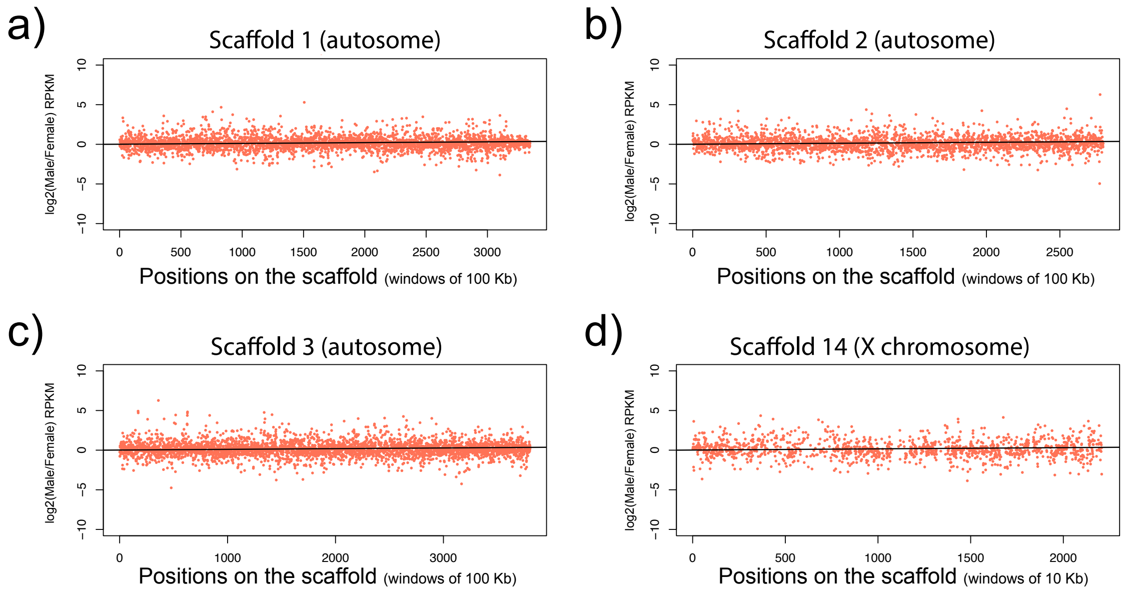

Supplementary figure 7. Dotplots showing the male-to-female expression ratio for autosomes S1, S2, and S3 (a-c), and also for the X chromosome (d). We observed that all chromosomes have balanced expression levels along their sequence.

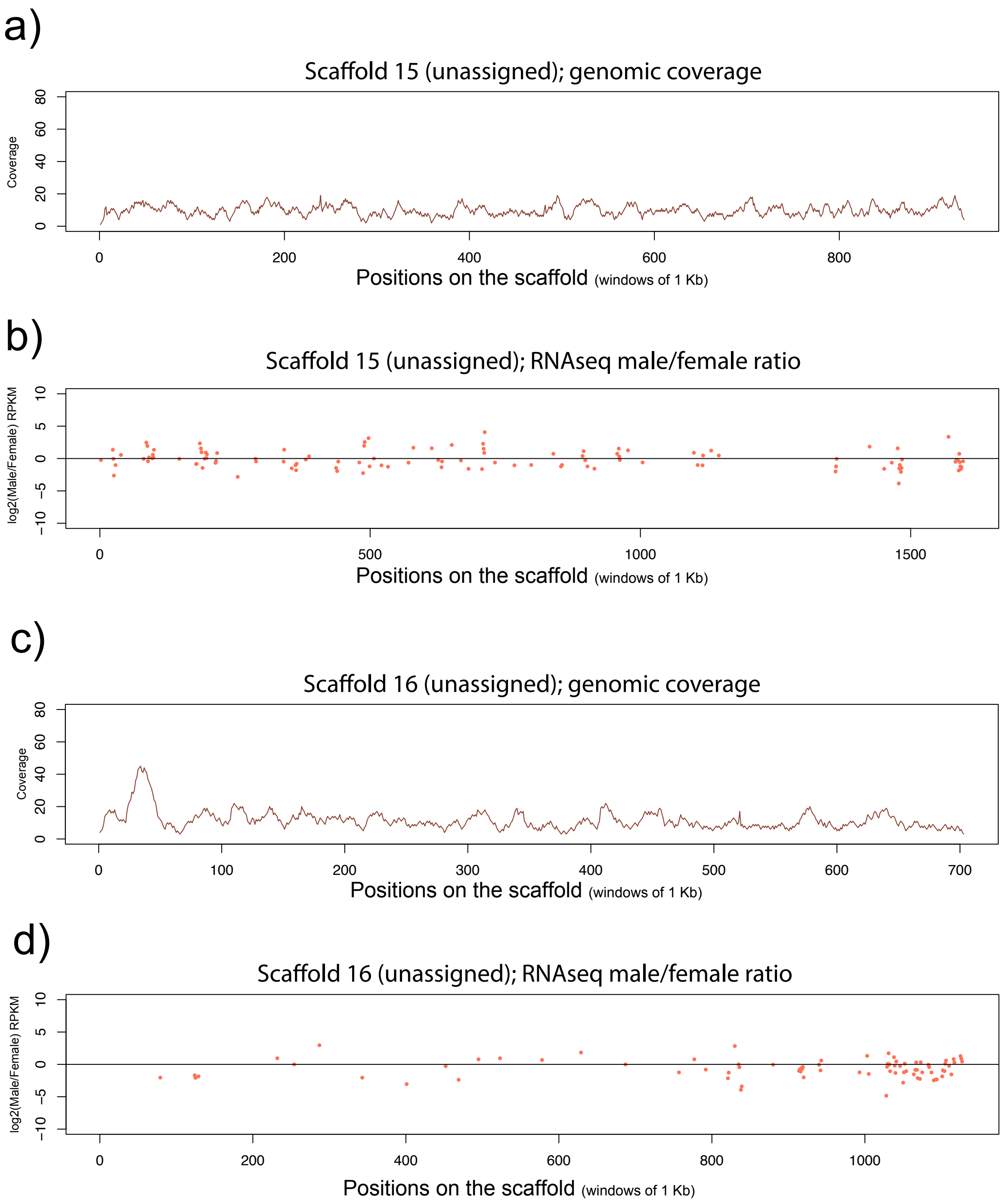

Supplementary figure 8. Dotplots showing the genomic read coverage for unassigned Scaffolds S15 (a) & S16 (c) and the male-to-female expression ratio for unassigned Scaffolds S15 (b) & S16 (d).
